# Intraspecific Differences in Biogeochemical Responses to Thermal Change in the Coccolithophore *Emiliania huxleyi*

**DOI:** 10.1371/journal.pone.0162313

**Published:** 2016-09-01

**Authors:** Paul G. Matson, Tanika M. Ladd, Elisa R. Halewood, Rahul P. Sangodkar, Bradley F. Chmelka, M. Debora Iglesias-Rodriguez

**Affiliations:** 1 Department of Ecology, Evolution, and Marine Biology, University of California Santa Barbara, Santa Barbara, California, United States of America; 2 Marine Science Institute, University of California Santa Barbara, Santa Barbara, California, United States of America; 3 Department of Chemical Engineering, University of California Santa Barbara, Santa Barbara, California, United States of America; University of Connecticut, UNITED STATES

## Abstract

The species concept in marine phytoplankton is defined based on genomic, morphological, and functional properties. Reports of intraspecific diversity are widespread across major phytoplankton groups but the impacts of this variation on ecological and biogeochemical processes are often overlooked. Intraspecific diversity is well known within coccolithophores, which play an important role in the marine carbon cycle via production of particulate inorganic carbon. In this study, we investigated strain-specific responses to temperature in terms of morphology, carbon production, and carbonate mineralogy using a combination of microscopy, elemental analysis, flow cytometry, and nuclear magnetic resonance. Two strains of the cosmopolitan coccolithophore *E*. *huxleyi* isolated from different regions (subtropical, CCMP371; temperate, CCMP3266) were cultured under a range of temperature conditions (10°C, 15°C, and 20°C) using batch cultures and sampled during both exponential and stationary growth. Results for both strains showed that growth rates decreased at lower temperatures while coccosphere size increased. Between 15°C and 20°C, both strains produced similar amounts of total carbon, but differed in allocation of that carbon between particulate inorganic carbon (PIC) and particulate organic carbon (POC), though temperature effects were not detected. Between 10°C and 20°C, temperature effects on daily production of PIC and POC, as well as the cellular quota of POC were detected in CCMP3266. Strain-specific differences in coccolith shedding rates were found during exponential growth. In addition, daily shedding rates were negatively related to temperature in CCMP371 but not in CCMP3266. Despite differences in rates of particulate inorganic carbon production, both strains were found to produce coccoliths composed entirely of pure calcite, as established by solid-state ^13^C and ^43^Ca NMR and X-ray diffraction measurements. These results highlight the limitations of the species concept and the need for a trait-based system to better quantify diversity within marine phytoplankton communities.

## Introduction

Marine phytoplankton are important components of the ocean carbon cycle that fix carbon dioxide via photosynthesis, enhance the concentration of organic matter, and export that matter to the deep ocean [1]. In each of the three major marine eukaryotic phytoplankton groups (diatoms, dinoflagellates, and coccolithophores), the species concept comprises genomic, morphological, and functional properties. Specifically, intraspecific diversity is widespread across groups of marine phytoplankton, with examples found in diatoms [2–4], coccolithophores [5, 6], and dinoflagellates [7]. However, despite the vast spectrum of functional traits within the species concept, intraspecific diversity has been largely overlooked when attempting to represent biological processes and their biogeochemical imprint in natural populations. Indeed, it has been argued that failing to account for intraspecific diversity underestimates the true scale of biodiversity and how it may be impacted by a changing climate [8]. A loss in diversity could lead to a shift in the relative abundance of strains with different properties, which is likely to alter ecosystem function and biogeochemistry [9].

Among the phytoplankton functional groups in the modern ocean, coccolithophores have received particular attention when studying the carbon cycle because, in addition to photosynthesis, cells also fix dissolved inorganic carbon by precipitating calcium carbonate plates (coccoliths). Unlike photosynthesis, calcification produces carbon dioxide as a byproduct. Thus, the ratio of particulate inorganic carbon (PIC) to particulate organic carbon (POC) production (producing versus consuming CO_2_, respectively) indicates whether phytoplankton production acts as a source (PIC:POC > 1.5) or sink (PIC:POC < 1.5) of carbon dioxide.

The coccolithophore *Emiliania huxleyi* (Lohm.) Hay and Mohler is a globally distributed species that is responsible for a significant proportion of biogenic calcite production in the world’s ocean [10–12]. Intraspecific variation is well recognized in *E*. *huxleyi* with strains showing diversity in genetic identity [5, 13], calcification [14, 15], and functional responses to environmental variation [16–18]. This species is well known for forming extensive seasonal blooms, primarily in the open ocean of high latitudes, although small-scale blooms have also been reported in coastal zones [19–22]. Unlike other coccolithophores, *E*. *huxleyi* produces multiple layers of coccoliths that can be shed during its life cycle [23]. Large quantities of shed coccoliths are visible from space due to their high reflectance properties, which enables the use of remote sensing to track these blooms and estimate the associated surface PIC [24]. In addition, laboratory experiments have shown that shed coccoliths are more efficiently incorporated into aggregates containing organic matter than larger foraminifera tests, increasing the sinking rate of these aggregates [25] and highlighting the importance of these blooms in carbon sequestration. Responses to environmental change are well studied within the coccolithophore community with a large proportion focused on assessing risks of ocean warming and acidification. Most studies investigating intraspecific diversity in functional responses to environmental pressure have focused on ocean acidification and nutrient availability [16–18]. Temperature appears to be important in modulating how *E*. *huxleyi* responds to other environmental stressors, such as acidification [26]. However, whether diverse responses to thermal alterations occur within the *E*. *huxleyi* species concept remains an open question.

In this study, we tested whether the observed intraspecific diversity in morphology and calcification extends to responses to thermal change in *E*. *huxleyi*. We used two strains isolated from two oceanographic regions with distinct temperature regimes, to test their physiology at 10, 15 and 20°C, which include temperatures within and outside their optimal growth. We quantified how temperature affected carbon production, morphology, coccolith detachment, and the types of calcium carbonate moeities in each strain using a combination of elemental analysis, scanning electron microscopy, flow cytometry, and nuclear magnetic resonance measurements.

## Materials and Methods

### Culturing conditions

**Fig 1:**
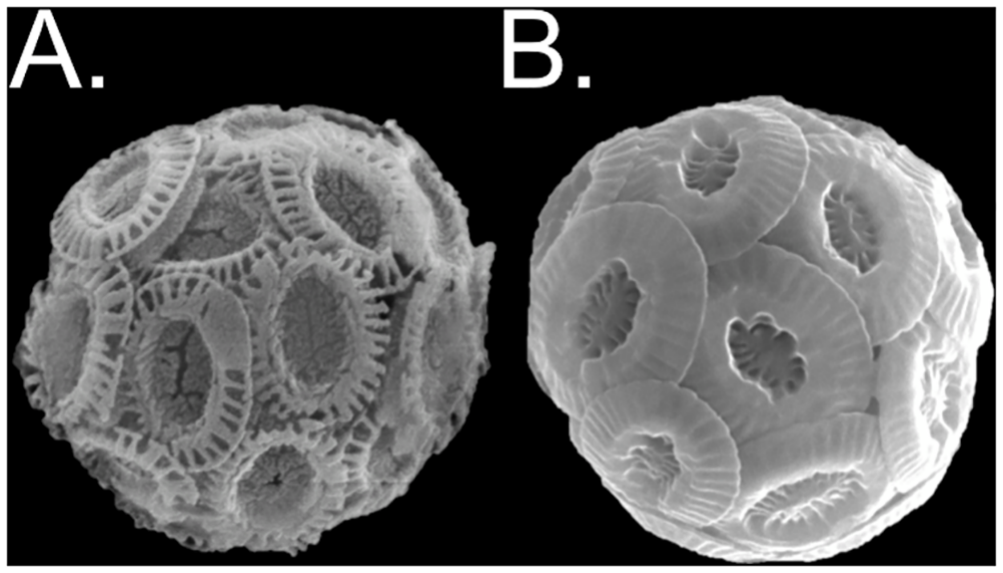
Representative images of *E*. *huxleyi*. Scanning electron microscopy images of cells representative of (A) the lower calcifying strain CCMP371 and (B) the higher calcifying strain CCMP3266. The malformed-looking coccoliths seen in CCMP371 were commonly seen under all treatment conditions. Image sizes are not to scale.

Two strains of *Emiliania huxleyi* (Lohm.) Hay and Mohler, CCMP371 and CCMP3266, were obtained from the National Center for Marine Algae and Microbiota at Bigelow Laboratory (Maine, USA). CCMP371 was isolated from the Sargasso Sea and CCMP3266 (strain synonym: RCC1216) was isolated from the Tasman Sea and have morphotypes displaying different levels of coccolith calcification (Fig 1). Cultures were maintained in modified f medium, containing seawater from the Santa Barbara Channel supplemented with 100 μM nitrate, 6.24 μM phosphate, and *f*/2 concentrations of trace metals and vitamins [27]. The resulting medium was filter-sterilized through 0.22 μm Steritop^™^ filtration units (EMD Millipore, USA) prior to cell inoculation. Cells were grown under a 12:12 h light:dark cycle at 140 μmol photons m^-2^ s^-1^ using EnviroGro^™^ T5 (Hyrdofarm, USA) white fluorescent lighting. Each day, cultures were gently swirled to suspend cells and their position was rotated underneath the lights.

Experiments were conducted sequentially through time, in which cells were grown at 20°C, 15°C, and 10°C (3266 only) in a temperature-controlled environmental room. Prior to initiating the experimental cultures, stocks of each strain were allowed to acclimate to each new temperature. Experimental cultures were inoculated with exponentially growing cells from these acclimated stocks at a concentration of 200 cells mL^-1^ into 16 L of culture media in a 20 L Nalgene^™^ polypropylene carboy. Aliquots (800 mL) of this culture were transferred into borosilicate flasks (1 L). One flask was opened repeatedly to estimate growth during each trial. Sampling of the remaining flasks occurred twice during exponential and twice during stationary phase.

### Cell density, growth rate, and nutrient utilization

Cell density was determined prior to each sampling event. Aliquots of undiluted cultures were used for cell counting using a hemocytometer. Triplicate samples were obtained during the exponential growth phase to calculate growth rate (μ) according to the relation:

$$\mu =\frac{{\text{(lnC}}_{\text{2}}{\text{-lnC}}_{\text{1}}\text{)}}{\Delta \text{t}}$$

where C_1_ and C_2_ refer to the initial and final cell densities and Δt refers to the number of days between samples.

Culture aliquots of 20 mL were filtered through 0.45 μm syringe filters and stored in glass scintillation vials at -20°C for seawater nutrient analyses. Samples were analyzed for nitrate + nitrite and phosphate using flow injection analysis (QuikChem 8000, Zellweger Analytics) in the UCSB Marine Science Institute Analytical Laboratory.

### Morphometric analysis of coccospheres

At each sampling time point, an aliquot of 1–3 mL was taken from one flask within each treatment and gently vacuum-filtered through a 13 mm membrane filter of 0.45 μm cellulose nitrate (MF^™^, EMD-Millipore) or 0.4 μm polycarbonate (Isopore, EMD-Millipore) for coccosphere size measurements. Filters were dried overnight at room temperature and stored in plastic Petri-slide cases. Each dried filter was affixed to a 12.7 mm aluminum pin stub using carbon conductive Pelco Tabs^™^ (Ted Pella, USA) and sputter-coated with gold for 300 s prior to scanning electron microscopy (SEM) analyses. These samples were examined under a Zeiss EVO 40 XVP scanning electron microscope at the Santa Barbara Museum of Natural History (CA, USA). Samples were viewed at 5,000x magnification and digital images were saved for analysis using ImageJ (http://imagej.nih.gov/ij/). For each sampling point, coccosphere diameters were measured along two axes, representing the maximum and minimum diameters for each individual (n = 50).

### Particulate carbon and nitrogen analyses

Aliquots of 100 mL were filtered onto 25 mm glass fiber filters (EMD-Millipore) and stored at -20°C for inorganic and organic carbon and nitrogen analyses. Filters were cut in half; one section was analyzed for total particulate carbon (TC) and nitrogen (TN) and the other section was acidified to remove any inorganic carbonates and then analyzed for particulate organic carbon (POC). Particulate inorganic carbon (PIC) was then calculated as the difference between TC and POC for each sample. Both analyses were conducted using the Dumas combustion method in an automated elemental analyzer (Model CE-440HA, Exeter Analytical) at the UCSB Marine Science Institute Analytical Laboratory. Daily PIC and POC production (*P*_*PIC*_ and *P*_*POC*_; biomass cell^-1^ day^-1^) were calculated as:

$$P_{PIC}{=\mu *\text{(pg PIC cell}}^{\text{-1}}\text{)}$$

$$P_{POC}{=\mu *\text{(pg POC cell}}^{\text{-1}}\text{)}$$

### Flow cytometry analysis

Aliquots of 9 mL were preserved by adding a 10x concentrated stock of formaldehyde/glutaraldehyde fixative and frozen at -20°C for further flow cytometry analysis. Detached coccolith to cell ratios were determined using a BD Influx Flow Cytometer (BD Biosciences) equipped with three lasers (355 nm, 488 nm, and 640 nm), a small particle detector, and polarization-sensitive detectors. Thawed experimental samples were thoroughly mixed and aliquoted into three 1-mL technical replicate samples. Cells were identified in the preserved samples by adding a nucleic acid stain (SYBR^®^ Green I; ThermoFisher Scientific) followed by a 15-min dark incubation. Lastly, quality-control beads (Ultra Rainbow Beads; Spherotech) were added as an internal reference immediately before running each sample on the flow cytometer. Samples were first analyzed for approximately one minute to stabilize the rate at which particles pass through the lasers (event rate), after which 50,000 events were recorded for each sample. After each sample analysis, the sample intake line was flushed with Milli-Q water for 5 minutes to prevent any carry-over of the previous sample. Analysis of the detached coccoliths:cell ratio was performed by gating cells based on the fluorescence signal from the nucleic acid stain (530/40-nm band pass filter) and coccoliths based on the polarized forward scatter signal and the absence of SYBR-I fluorescence using the cell sorter’s software program (BD FACS Sortware). Daily coccolith detachment rates (*D*_*liths*_, coccoliths cell^-1^ day^-1^) were calculated as:

$$D_{liths}=\;\mu *\frac{\text{coccoliths}}{\text{cells}}$$

### Solid-state nuclear magnetic resonance spectroscopy

Large volume cultures (~14 L) of strains CCMP371 and CCMP3266 were used to produce sufficient CaCO_3_ for solid-state nuclear magnetic resonance (NMR) analyses. Each strain was cultured in two 10-L polycarbonate vessels in a modified *f* medium containing 100 μM nitrate, 6.24 μM phosphate, and *f*/2 concentrations of trace metals and vitamins [27]. Cultures were maintained at 20°C under a 12:12 h light:dark cycle at 140 μmol photons m^-2^ s^-1^ using EnviroGro^™^ T5 (Hyrdofarm, USA) white fluorescent lighting. Cultures were gently swirled to re-suspend cells each day until dense cultures were obtained, either at or before the stationary growth phase. Cells were harvested through a combination of gravitational settling and centrifugation to remove as much culture media as possible. Samples were collected in 15 mL polypropylene tubes (Falcon) and stored at -80°C overnight. Frozen samples were then lyophilized to remove any remaining water and stored sealed at room temperature until NMR analyses.

Solid-state ^13^C magic-angle-spinning (MAS) nuclear magnetic resonance (NMR) measurements were conducted at 25°C using an 11.7 T Bruker AVANCE-II NMR spectrometer at the Central Facilities of the UCSB Materials Research Laboratory, operating at frequencies of 500.00 and 125.73 MHz for ^1^H and ^13^C, respectively, and using a 4-mm H-X-Y triple-resonance MAS probehead. One-dimensional (1D) single-pulse ^13^C MAS NMR spectra were acquired using a π/2 pulse length of 5 μs, 10 kHz MAS, with SPINAL-64 ^1^H decoupling (2.5 μs ^1^H π/2 pulse) [28], and a recycle delay of 7200 s (2 h). The long recycle delays allow thermal re-equilibration of the carbonate ^13^C magnetization between successive scans, due to the very long nuclear spin-lattice (*T*_1_) relaxation times (ca. 40 min) [29] of the ^13^C carbonate moieties, which reflect crystalline environments. The isotropic ^13^C chemical shifts were referenced to tetramethylsilane using tetrakis(trimethylsilyl)silane [((CH_3_)_3_Si)_4_Si] as a secondary standard [30]. Solid-state ^43^Ca MAS NMR measurements were conducted at 25°C using a 19.6 T Bruker DRX NMR spectrometer at the U.S. National High Magnetic Field Laboratory, Tallahassee, Florida, operating at frequencies of 830.00 and 55.97 MHz for ^1^H and ^43^Ca, respectively, and using a custom-built 7-mm single-resonance MAS probehead, especially designed for low-*γ* nuclei. 1D single-pulse ^43^Ca MAS NMR spectra were acquired using a π/2 pulse length of 4 μs, 5 kHz MAS, and a recycle delay of 0.5 s. The ^43^Ca shifts were referenced to a 1 M aqueous solution of CaCl_2_ [31].

### Statistical analyses

Data were analyzed using several different statistical tests to examine potential effects of strain, temperature, and growth phase on physiological and morphological parameters. All analyses were performed using R software (version 3.1.2). For each test, data were first examined for heterogeneity and normality of residuals. Data that met the assumptions for parametric testing were analyzed using either a one-, two-, or three-way ANOVA with Type II Sums of Squares to account for unbalanced sample sizes [32]. When appropriate, *posthoc* tests, either Tukey or Multiple Comparisons (R packages ‘agricolae’ and ‘pgirmess’ respectively), were used to identify highly significant differences between treatments. Data for coccosphere diameter and coccolith:cell ratios failed to meet assumptions for parametric ANOVA and were instead analyzed using generalized linear models (GLM) fit by a gamma distribution (R package ‘nlme’), which are appropriate for continuous data with values greater than zero [33]. Logarithmic or inverse linking functions to satisfy assumptions of homogeneity of residuals. Model parameters were selected based on minimizing the Akaike Information Criterion (AIC). The predictor variables of phase and strain were treated as categorical while temperature was treated as continuous. The significance of predictor variables within the selected model was tested using analysis of deviance with an *F*-test.

## Results

### Growth rate and nutrient utilization

Growth rates (μ) in both strains increased at warmer temperatures with CCMP3266 exhibiting higher growth rates at both 15 and 20°C. In CCMP3266, growth rates were 0.40, 0.74, and 1.10 d^-1^ at 10, 15, and 20°C, respectively. In CCMP371, growth rates were 0.42 and 0.77 d^-1^ at 15 and 20°C, respectively. During the exponential growth phase, cells were nutrient-replete in all cultures, with nitrate + nitrite and phosphate ranging from 83.2 to 103.3 μM and 2.1 to 3.2 μM, respectively (S1 Fig). During the stationary growth phase, all cells were nutrient limited, with phosphate levels below the method detection limit (0.1 μM) (S1 Fig).

### Differences in inorganic and organic carbon content between strains

**Fig 2:**
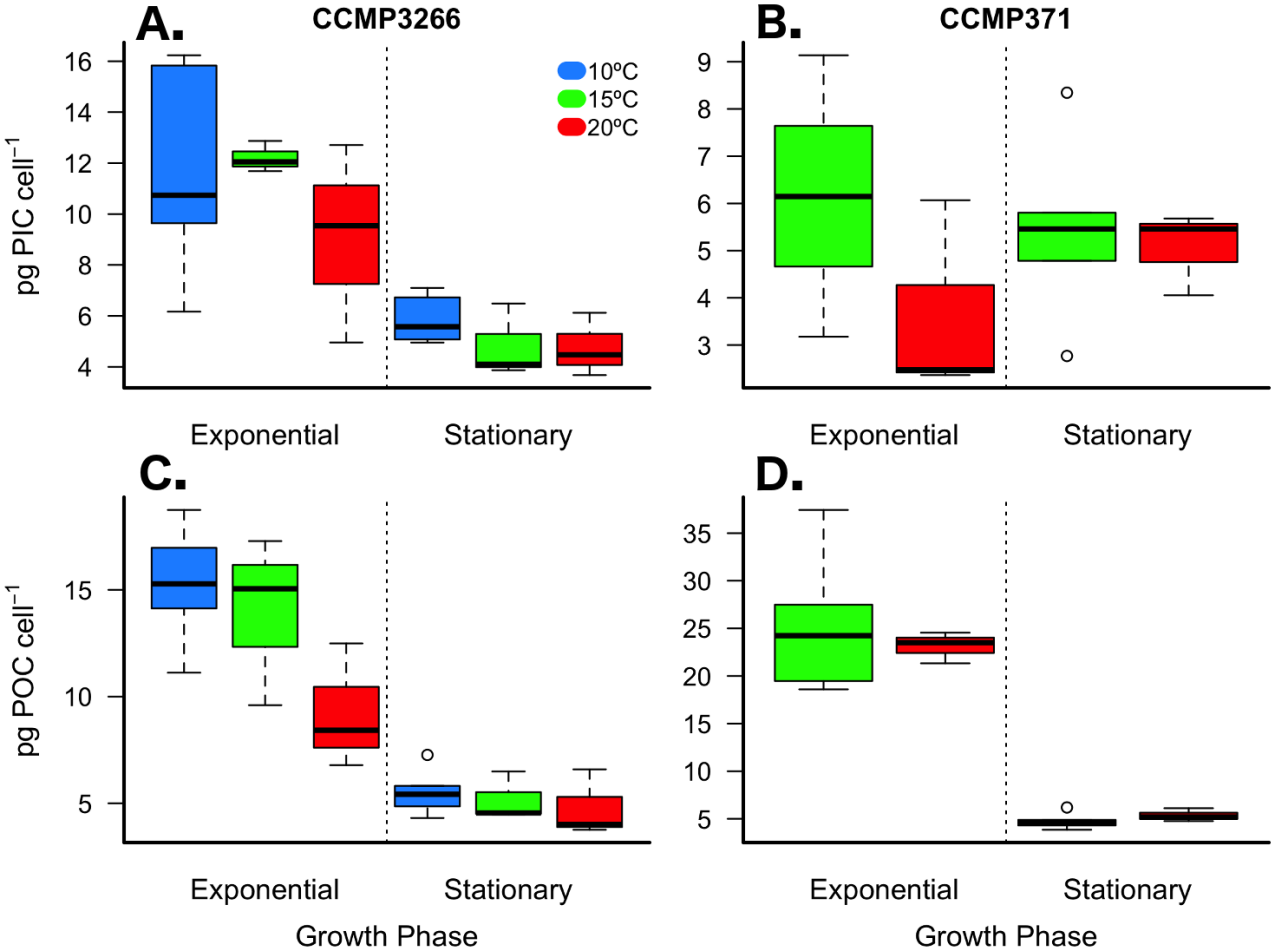
Particulate inorganic (PIC) and organic carbon (POC) content per cell. *E*. *huxleyi* strains CCMP3266 (A & C) and CCMP371 (B & D) cultured under multiple temperature conditions during exponential and stationary growth phases. Thick black line in boxplot represents median value for each experimental treatment.

The two strains tested showed contrasting PIC and POC cellular quotas when compared at 15 and 20°C. Overall, these two strains had similar cellular levels of TC (inorganic + organic) (Table 1; S1 Table) but contrasting allocation of inorganic and organic forms. In general, CCMP3266 appeared to be a higher calcifying strain compared to CCMP371, which fixed more organic carbon (Fig 2). During exponential phase, PIC cell^-1^ in CCMP3266 was 1.9 to 2.5 times greater than in CCMP371 (Fig 2; Table 1; S1 Table). Although no effect of temperature was detected, a large decrease in PIC cell^-1^ was observed between growth phases of CCMP3266 that was not evident in CCMP371 based on the significant interaction between strain and phase (Fig 2; Table 1; S1 Table). Organic carbon content (POC cell^-1^) in CCMP371 was 1.7 to 2.5 times greater than in CCMP3266 (Fig 2; Table 1; S1 Table). No temperature effect was detected, but a large (~ 5-fold) difference in POC cell^-1^ was observed in CCMP371 between growth phases (Fig 2; Table 1; S1 Table).

**Table 1:** Physiological parameters of *Emiliania huxleyi* strains CCMP371 and CCMP3266.

**Strain**	**°C**	**Phase**	**μ**	**TC**	***P***_***TC***_	**PIC:POC**
			**(d**^**-1**^**)**	**(pg cell**^**-1**^**)**	**(pg cell**^**-1**^ **d**^**-1**^**)**	
CCMP371	20	E	0.79 ± 0.00	26.77 ± 0.80	21.12 ± 0.63	0.16 ± 0.11
		S	–	10.42 ± 1.43	–	0.95 ± 0.13
	15	E	0.42 ± 0.02	30.45 ± 7.59	12.74 ± 2.58	0.25 ± 0.09
		S	–	10.18 ± 2.55	–	1.13 ± 0.23
CCMP3266	20	E	1.08 ± 0.06	18.30 ± 6.73	19.57 ± 6.24	0.96 ± 0.21
		S	–	9.55 ± 2.79	–	1.01 ± 0.10
	15	E	0.71 ± 0.03	26.18 ± 4.28	18.48 ± 2.33	0.93 ± 0.29
		S	–	9.99 ± 2.59	–	0.92 ± 0.07
	10	E	0.40 ± 0.01	26.81 ± 5.85	10.76 ± 2.03	0.75 ± 0.21
		S	–	11.35 ± 1.79	–	1.07 ± 0.13
**Strain**	**°C**	**Phase**	**PIC**	***P***_***PIC***_	**POC**	***P***_***POC***_
			**(pg cell**^**-1**^**)**	**(pg cell**^**-1**^ **d**^**-1**^**)**	**(pg cell**^**-1**^**)**	**(pg cell**^**-1**^ **d**^**-1**^**)**
CCMP371	20	E	3.64 ± 2.11	2.87 ± 1.66	23.13 ± 1.64	18.25 ± 1.29
		S	5.06 ± 0.88	–	5.36 ± 0.69	–
	15	E	6.15 ± 2.98	2.56 ± 1.14	24.29 ± 4.94	10.18 ± 1.59
		S	5.44 ± 1.80	–	4.74 ± 0.80	–
CCMP3266	20	E	9.07 ± 3.90	9.68 ± 3.74	9.23 ± 2.94	9.90 ± 2.64
		S	4.76 ± 1.25	–	4.79 ± 1.57	–
	15	E	12.20 ± 0.61	8.64 ± 0.32	13.98 ± 3.95	9.84 ± 2.46
		S	4.82 ± 1.45	–	5.17 ± 1.14	–
	10	E	11.56 ± 3.88	4.63 ± 1.44	15.25 ± 2.60	6.13 ± 0.87
		S	5.84 ± 0.92	–	5.52 ± 1.01	–
**Strain**	**°C**	**Phase**	**PON**	***P***_***PON***_	**TC:TN**	
			**(pg cell**^**-1**^**)**	**(pg cell**^**-1**^ **d**^**-1**^**)**		
CCMP371	20	E	6.17 ± 1.96	4.87 ± 1.54	5.02 ± 3.05	
		S	0.9 ± 0.12	–	10.64 ± 0.06	
	15	E	1.59 ± 0.56	0.67 ± 0.23	6.23 ± 0.91	
		S	0.68 ± 0.10	–	12.28 ± 0.79	
CCMP3266	20	E	1.32 ± 0.39	1.42 ± 0.34	11.16 ± 3.31	
		S	0.73 ± 0.24	–	13.04 ± 0.27	
	15	E	1.81 ± 0.41	1.28 ± 0.24	14.87 ± 2.98	
		S	0.79 ± 0.18	–	13.06 ± 0.44	
	10	E	2.98 ± 0.92	1.20 ± 0.34	10.09 ± 1.65	
		S	0.85 ± 0.16	–	13.34 ± 0.17	

E, exponential; S, stationary; μ, growth rate; PIC, particulate inorganic carbon; *P*_*PIC*_, PIC production; POC, particulate organic carbon; *P*_*POC*_, POC production; PON, particulate organic nitrogen; *P*_*PON*_, PON production; TC, total carbon (PIC+POC); TN, total nitrogen (see methods for description). Values are shown as mean ± standard deviation where n = 3, except CCMP3266 at 10°C where n = 6.

**Fig 3:**
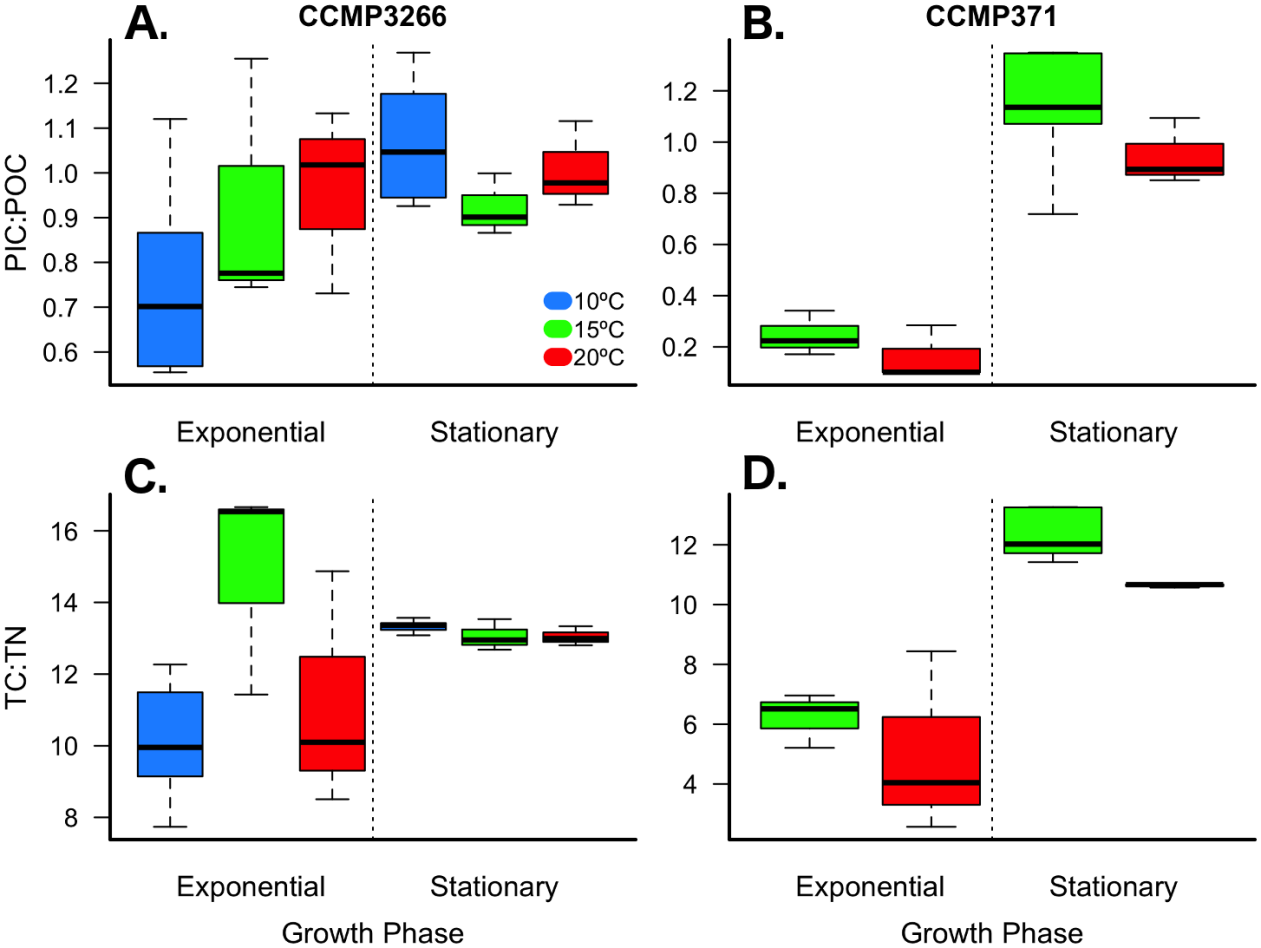
Ratios of particulate inorganic (PIC) to organic carbon (POC) and total carbon (TC) to total nitrogen (TN). *E*. *huxleyi* strains CCMP3266 (A & C) and CCMP371 (B & D) cultured under multiple temperature conditions during exponential and stationary growth phases. Thick black line in boxplot represents median value for each experimental treatment.

The distinct compartmentalization of carbon between theses strains is reflected in the PIC:POC ratio, which differed significantly across strains and growth phases, but not with respect to temperature (Fig 3; Table 1; S1 Table). PIC:POC during exponential growth was 3.7 to 6.0 times lower in CCMP371 than in CCMP3266, whereas there was no difference between the strains during stationary growth (Fig 3; Table 1; S1 Table). The amount of TC was comparable but the ratio of TC:TN differed across strains, temperatures, and growth phases (Fig 3, Table 1; S1 Table). During exponential growth, the ratio of TC:TN in CCMP3266 was > 2 times greater than that of CCMP371.

**Fig 4:**
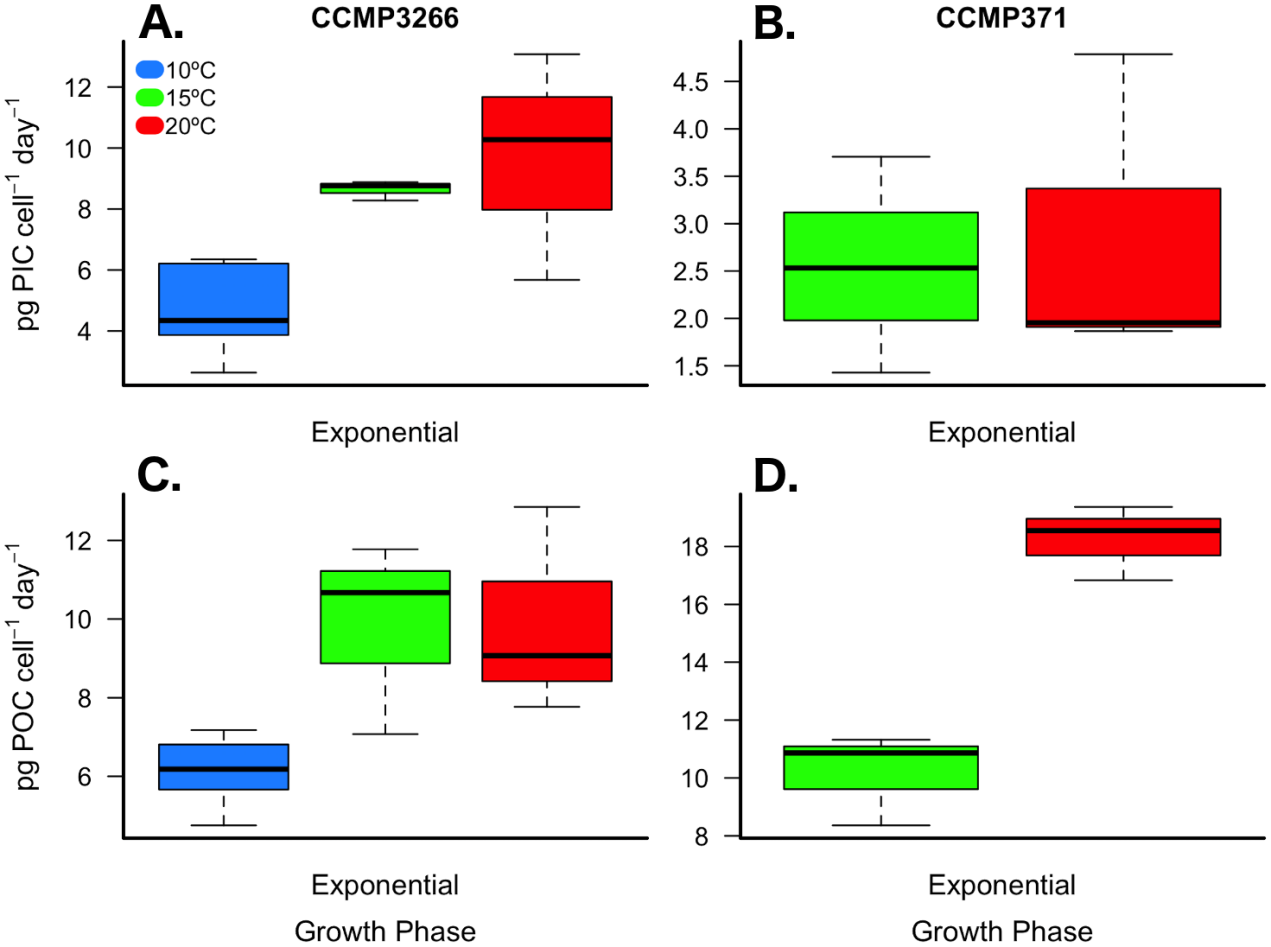
Daily production of particulate inorganic (PIC) and organic carbon (POC) content. *E*. *huxleyi* strains CCMP3266 (A & C) and CCMP371 (B & D) cultured under multiple temperature conditions during exponential growth. Thick black line in boxplot represents median value for each experimental treatment.

Daily production of inorganic carbon during exponential growth in CCMP3266 was 3.3 to 3.4 times greater than CCMP371 but no effect of temperature was detected (Fig 4; Table 1; S2 Table). Daily organic carbon production was greater in CCMP371 at 20°C, but was similar to CCMP3266 at 15°C (Fig 4; Table 1; S2 Table).

### Temperature dependence of inorganic and organic carbon production

Carbon content and production across a 10°C temperature range (10, 15, and 20°C) were analyzed separately for CCMP3266 during exponential and stationary growth phases. Daily total particulate carbon production during exponential growth was 45% lower at 10°C compared to 20°C (Table 1; one-way ANOVA: F_(2,7)_ = 8.51, *p* = 0.008). POC cell^-1^ showed a significant response to temperature during exponential growth with values increasing by 65% and 51% at 10°C and 15°C, respectively, when compared to 20°C (Fig 2; Table 1; S3 Table). However, daily POC production was 39% lower at 10°C compared to 20°C (Fig 2; Table 1; one-way ANOVA: F_(2,7)_ = 6.33, *p =* 0.019). While PIC cell^-1^ remained unaffected by changes in temperature during exponential growth (Fig 2; Table 1; S3 Table), daily PIC production (PIC cell^-1^ day^-1^) decreased by 53% at 10°C compared to 20°C (Fig 4; Table 1; one-way ANOVA: F_(2,7)_ = 7.38, *p* = 0.013). Growth phase had a significant effect on all dependent variables, though a significant interaction between phase and temperature was detected for the TC:TN ratio, likely due to the non-linear response to temperature during exponential growth (Fig 3; Table 1; S3 Table).

### Differences in coccosphere size and shape between strains

Coccosphere size was compared between each strain based on maximum and minimum measurements of each cell (n = 50; Table 2). Maximum size decreased inversely with temperature in both strains, shrinking by ~7% at 20°C in both strains, but only during the exponential phase. An inverse-link GLM of maximum coccosphere diameter found significant effects of phase, strain, and temperature (F_1,798_ = 212.4, *p* < 0.001; F_1,797_ = 4.7, *p* = 0.030; F_1,796_ = 26.1, *p* < 0.001), as well as significant interactions between phase:strain, and phase:temperature (F_1,795_ = 36.5, *p* < 0.001; F_1,794_ = 13.3, *p* < 0.001; respectively). Minimum size also decreased inversely with temperature during the exponential phase only, decreasing ~4 and 7% at 20°C in CCMP371 and CCMP3266, respectively. An inverse-link GLM of maximum coccosphere diameter found significant effects of phase and temperature (F_1,798_ = 224.2, *p* < 0.001; F_1,796_ = 16.5, *p* = < 0.001, respectively) but not strain (F_1,797_ = 2.5, *p* = 0.11), as well as significant interactions between phase:strain, phase:temperature, and strain:temp (F_1, 795_ = 18.5, *p* < 0.001; F_1,794_ = 11.1, *p* < 0.001; F_1,793_ = 8.8, *p* = 0.003; respectively).

**Table 2:** Coccosphere morphological parameters of *Emiliania huxleyi* strains CCMP371 and CCMP3266.

Strain	Phase	°C	Mean Maximum Diameter	Mean Minimum Diameter	Mean Symmetry
CCMP371	E	15	6.69 ± 0.82	5.85 ± 0.81	1.15 ± 0.09
		20	6.24 ± 0.84	5.61 ± 0.80	1.12 ± 0.07
	S	15	5.45 ± 0.56	4.82 ± 0.54	1.13 ± 0.09
		20	5.50 ± 0.49	4.96 ± 0.54	1.11 ± 0.08
CCMP3266	E	10	6.93 ± 0.83	6.39 ± 0.69	1.09 ± 0.07
		15	6.28 ± 0.67	5.85 ± 0.55	1.07 ± 0.06
		20	5.85 ± 0.71	5.40 ± 0.60	1.08 ± 0.06
	S	10	6.35 ± 0.72	5.79 ±0.60	1.10 ± 0.08
		15	5.73 ± 0.66	5.22 ± 0.54	1.10 ± 0.07
		20	5.60 ± 0.63	5.06 ± 0.57	1.11 ± 0.07

E, exponential; S, stationary. Values are reported as mean ± s.d. where n = 50.

When strain CCMP3266 was analyzed separately across all temperature treatments, maximum coccosphere diameter decreased inversely with temperature by ~16% between 10°C and 20°C in the exponential phase, but only ~12% during the stationary phase. An inverse-link GLM of maximum coccosphere diameter found significant effects of phase, temperature (F_1,598_ = 210.3, *p* < 0.001; F_1,597_ = 52.6, *p* < 0.001; respectively), as well as significant interaction between phase:temperature (F_1, 596_ = 8.2, *p* = 0.004). Minimum coccosphere diameter decreased inversely with temperature by ~14% between 10°C and 20°C with not significant difference between the growth phases. An inverse-link GLM of maximum coccosphere diameter found significant effects of phase and temperature (F_1,498_ = 126.6, *p* < 0.001; F_1,497_ = 127.1, *p* < 0.001; respectively). Cell symmetry in CCMP3266 did not show any significant differences between temperatures or growth phases.

### Coccolith shedding rate

**Fig 5:**
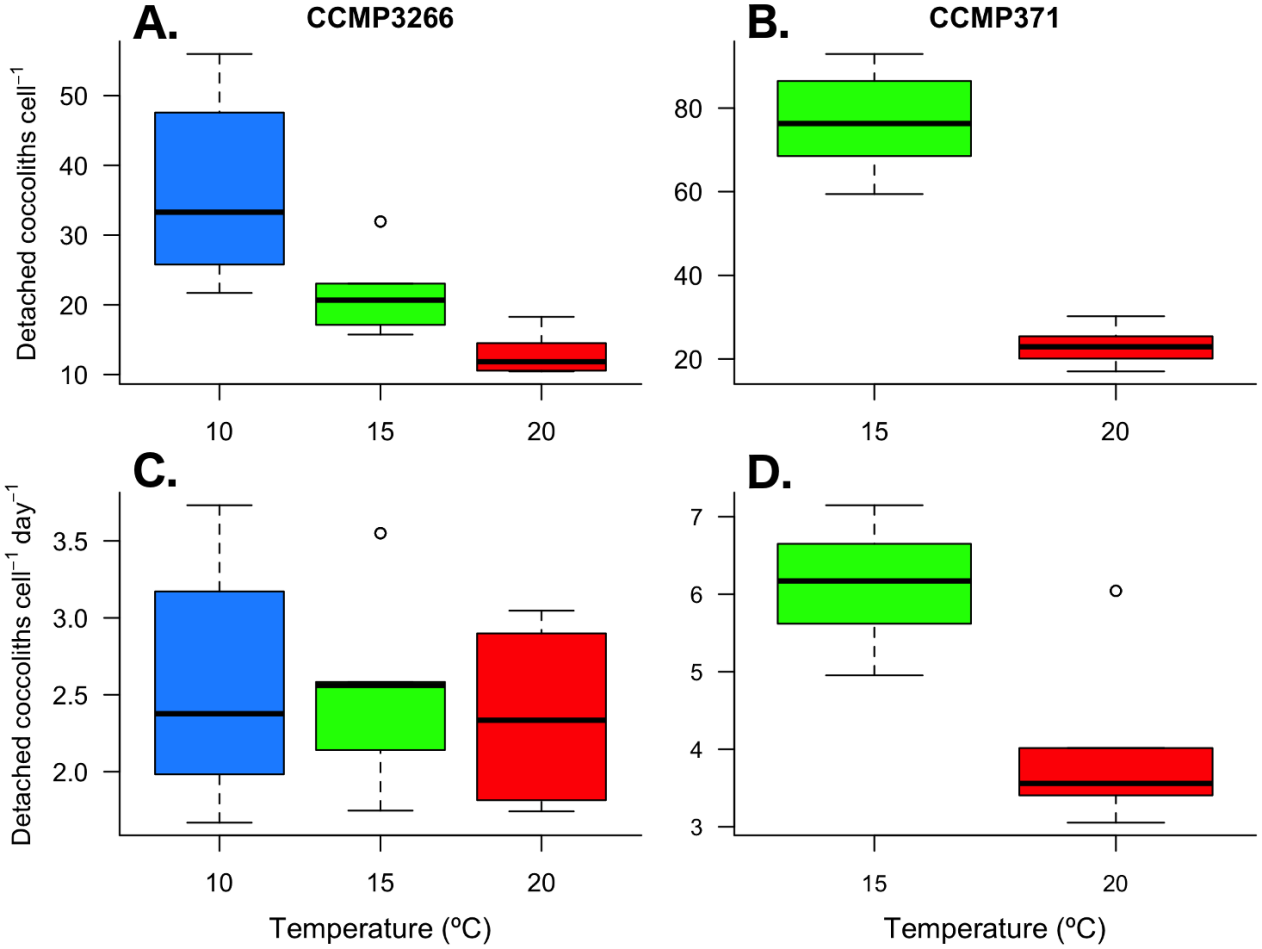
Detached coccoliths per cell and daily coccolith detachment rates. *E*. *huxleyi* strains CCMP3266 (A & C) and CCMP371 (B & D) cultured under multiple temperature conditions during exponential growth. Thick black line in boxplot represents median value for each experimental treatment.

Coccolith shedding differed between the two strains, both in terms of detached coccoliths cell^-1^ and the daily rate of coccolith detachment (*D*_liths_) during exponential growth. Flow cytometry showed that the number of detached coccoliths in CCMP371 (76.7 ± 11.5 and 23.1 ± 4.3 coccoliths cell^-1^ at 15°C and 20°C, respectively) was 3.7 and 1.8 times higher than in CCMP3266 (21.0 ± 5.1 and 12.9 ± 2.9 coccoliths cell^-1^ (Fig 5A and 5B). A log-link GLM of the number of detached coccoliths cell^-1^ found a significant effect of strain and temperature (F_1,70_ = 483.9, *p* < 0.001; F_1,69_ = 293.5, *p* < 0.001; respectively), as well as an interaction between strain:temperature F_1,68_ = 54.9, *p* < 0.001). After accounting for growth rate (μ), the calculated daily detachment rate of coccoliths in CCMP371 (32.2 ± 4.8 and 17.8 ± 3.3 coccoliths cell^-1^ day^-1^ at 15°C and 20°C, respectively) was 2.1 and 1.3 times higher than in CCMP3266 (15.6 ± 3.7 and 14.2 ± 3.2 coccoliths cell^-1^ day^-1^) (Fig 5C and 5D). Further, while the daily coccolith detachment rate was temperature sensitive in CCMP371, this was not seen in CCMP3266 (Fig 5C and 5D). A log-link GLM of the daily rate of coccolith detachment (*D*_liths_) found a significant effect of strain and temperature (F_1,70_ = 115.8, *p* < 0.001; F_1,69_ = 50.3, *p* < 0.001; respectively), as well as an interaction between strain:temperature (F_1,68_ = 27.4, *p* < 0.001).

### Local structures of CaCO_3_ in coccoliths

**Fig 6:**
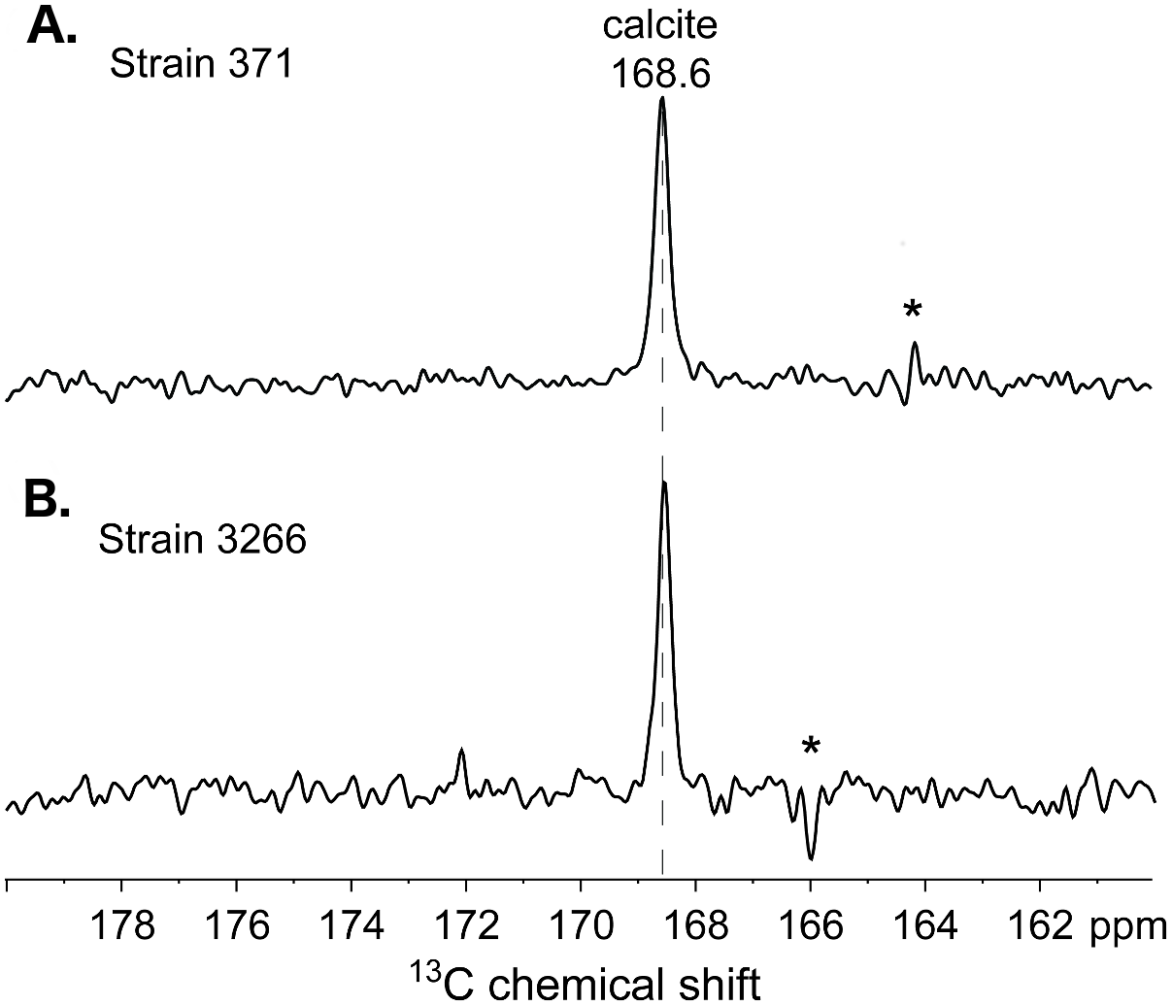
Solid-state ^13^C NMR. 1D single-pulse ^13^C MAS NMR spectra of *E*. *huxleyi* strains (A) CCMP371 and (B) CCMP3266 acquired at 11.7 T, 25°C, and 10 kHz MAS. The asterisks indicate a frequency artifact corresponding to the ^13^C excitation pulse used in the experiment.

The atomic-level structures and compositions of inorganic carbonates produced by CCMP371 and CCMP3266 were established by using solid-state ^13^C NMR measurements, which are sensitive to the atomic bonding environments near ^13^C moieties, including those lacking long-range (>10 nm) structural order. Both *E*. *huxleyi* strains examined here were composed of organic moieties (*e*.*g*., amino acids, carbohydrates) in addition to the inorganic carbonates, both of which contain ^13^C in low (1.1%) natural isotopic abundance. Compared to the predominant inorganic calcium carbonate fractions, the dilute (< 5 wt%) and diverse organic moieties in both CCMP371 and CCMP3266 are below the detection limit of the single-pulse ^13^C MAS NMR measurements used here. Such measurements, nevertheless, enable the detection and identification of the carbonate ^13^C moieties in the coccolithophore biominerals, based on their narrow and distinct ^13^C isotropic chemical shifts. Single-pulse ^13^C MAS NMR spectra (Fig 6) of strains CCMP371 and CCMP3266 both exhibit only one ^13^C signal at 168.6 ppm, which corresponds to local ^13^C environments in calcite [34, 35]. The narrow linewidths (~0.3 ppm full-width-half-maximum, fwhm) of the signals reflect a narrow distribution of ^13^C environments, consistent with the long-range crystalline order of calcite, as established by complementary X-ray diffraction analyses (S2 Fig). This indicates that calcite is the primary ^13^C component in the *E*. *huxleyi* biominerals, within the detection limits (< 5 wt%) of the measurements.

**Fig 7:**
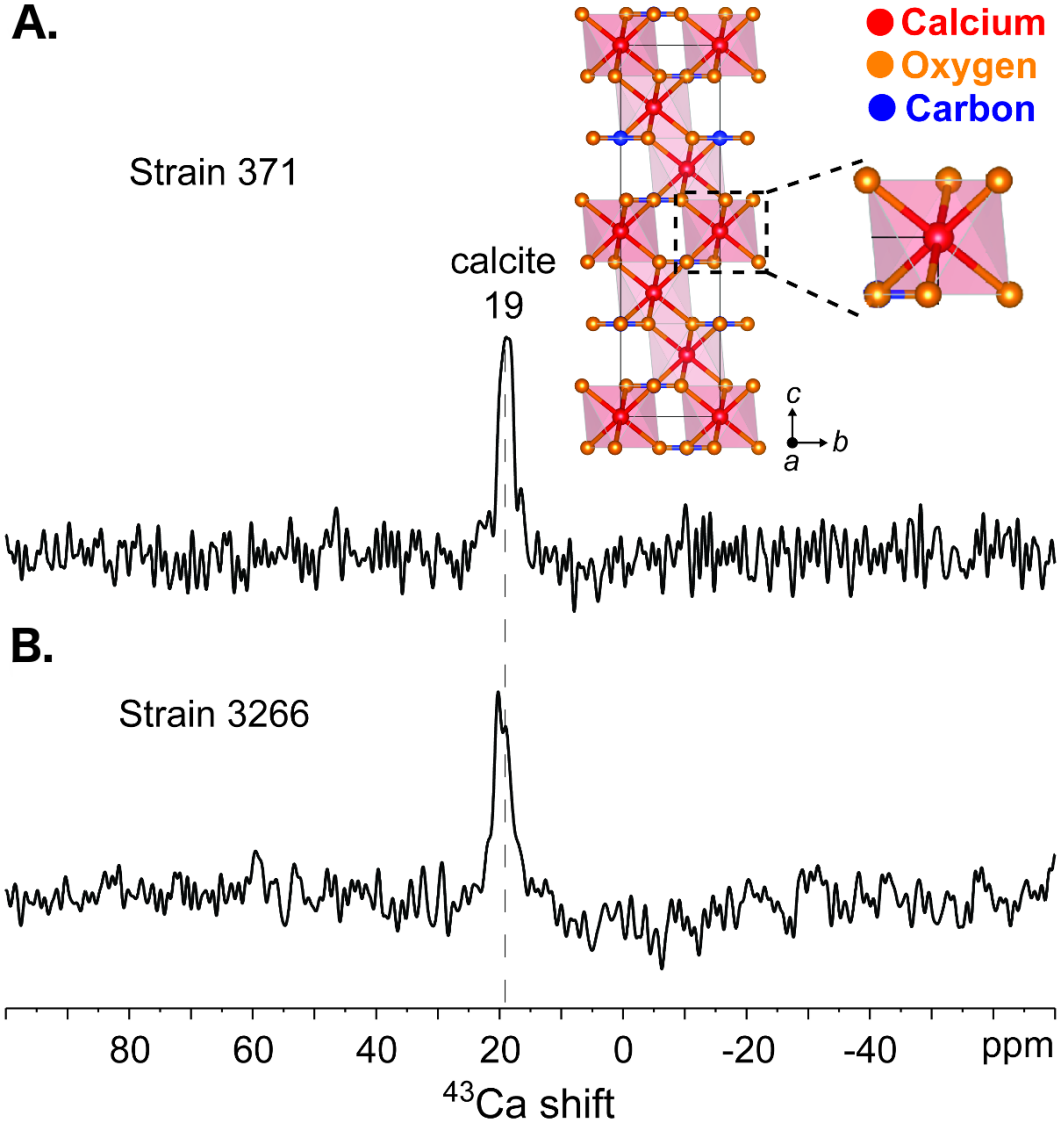
Solid-state ^43^Ca NMR. 1D single pulse ^43^Ca MAS NMR spectra of *E*. *huxleyi* strains (A) CCMP371 and (B) CCMP3266 acquired at 19.6 T, 25°C, 5 kHz MAS. The spectra were recorded over total measurement times of (A) 85 h and (B) 18 h. The inset in (A) is a schematic diagram of the hexagonal unit cell of calcite showing the six-fold coordination environments of the calcium atoms (red), along with the oxygen (orange) and carbon (blue) atoms.

In addition, the local atomic environments of calcium cations in the coccolithophore biominerals were directly probed by using solid-state ^43^Ca NMR measurements, which provide complementary information to the ^13^C NMR analyses. Compared to ^13^C nuclei (1.1% natural isotopic abundance, spin-1/2), the NMR-active isotope of calcium, ^43^Ca, is quadrupolar (spin-7/2), has a lower gyromagnetic ratio (*γ* = -1.803 x 10^7^ rad.s^-1.^T^-1^), and is present in extremely dilute quantities (0.135% natural isotopic abundance). This results in low sensitivity and often prohibitively long measurement times that present challenges for the detection of signals from ^43^Ca moieties [36]. Nevertheless, such challenges can be overcome, in part, by using very high magnetic fields (19.6 T), which yield enhanced signal sensitivity and mitigate the effects of quadrupolar interactions, resulting in improved ^43^Ca spectral resolution. Solid-state 1D single-pulse ^43^Ca NMR spectra (Fig 7) acquired at 19.6 T of strains CCMP371 and CCMP3266 both exhibit a ^43^Ca signal at 19 ppm. Based on previous solid-state ^43^Ca NMR studies of synthetic and naturally occurring calcium carbonates and related inorganic oxides of calcium [31, 37], the ^43^Ca signal at 19 ppm is attributable to calcite in the coccolithophore biominerals. In addition, the narrow ^43^Ca line-widths (ca. 3 ppm fwhm; Fig 7) reflect Ca^2+^ cation sites that are well ordered at the atomic-level. This is consistent with the near-octahedral symmetry (Fig 7a, inset) of the single type of six-coordinate ^43^Ca site in calcite. Notably, only the ^43^Ca signal at 19 ppm from calcite is observed in the single-pulse ^43^Ca spectra, even after extensive signal averaging (> 1 x 10^5^ scans), which indicates that there are no detectable quantities of other ^43^Ca moieties. Overall, the natural abundance ^13^C and ^43^Ca NMR establish the presence of calcite in the carbonate biominerals in strains CCMP371 and CCMP3266.

## Discussion

### Emiliania huxleyi strain variability in carbon fixation

Coccolithophores represent an important component of the marine carbon cycle, contributing up to 80% of calcification in the surface ocean [11, 38, 39]. *Emiliania huxleyi* is the most abundant, cosmopolitan, and ecologically important coccolithophore species and bears a great deal of strain diversity. In this study, we found that total carbon production did not differ significantly in response to temperature or between strains within each growth phase. However, strains allocated particulate carbon production differently between inorganic and organic forms. Specifically, during exponential growth, 80 to 86% of the particulate carbon produced by CCMP371 was in the organic form. In contrast, POC in CCMP3266 accounted for only 50 to 57% of its total particulate carbon. Many studies report on strain-specificity in carbon production, but comparisons between strains as a function of culturing conditions can be difficult because each laboratory uses different conditions (light, nutrients, temperature). For example, values from the literature show POC cell quotas ranging from 6 to 16.8 pg cell^-1^ in CCMP371 [40, 41], 5.7 to 16 pg cell^-1^ in CCMP3266 [42, 43], and 5 to 26.4 pg cell^-1^ in another high calcifying *E*. *huxleyi* strain, NZEH [44, 45]. Cell quotas of PIC have been shown to range from 2 to 9.3 pg cell^-1^ in CCMP371 [40, 41], 4.8 to 12 pg cell^-1^ in CCMP3266 [42, 46], and 3 to 24 pg cell^-1^ in NZEH [44, 45]. Experiments that test multiple strains under a uniform set of culturing conditions offer an ideal approach to conduct intraspecific comparisons (e.g., [16–18, 47]).

### Temperature adaptation within species

Temperature is an important environmental parameter that determines the distribution and performance of marine organisms. Strain-specific differences in growth rates are well documented for *E*. *huxleyi*, often reflecting the thermal ecotype from which a strain was originally isolated [48]. Both strains tested in this study showed a positive relationship between growth and temperature, though CCMP3266, which originates from a temperate south Pacific latitude (42.3°S) displayed higher growth rates than the subtropical strain CCMP371 (32°N). The in-situ temperature at the time of isolation of CCMP371 is unknown, though the National Center for Marine Algae and Microbiota reports the range to be between 17 and 26°C, and this strain showed an 83% increase in growth from 15 to 20°C. The other strain, CCMP3266, which was isolated from waters at ~11°C [16], displayed a comparable increase in growth rate (85%) when temperature increased from 10 to 15°C but growth rate went up by only 43% when the temperature increased from 15 to 20°C. Growth rates of CCMP371 at 20°C in this study were similar to those seen by Feng et al. [40] at the same temperature in semi-continuous cultures (μ ≈ 0.58–0.72) though lower than those reported at 17°C in continuous cyclostat cultures (μ = 0.93–1.26; [41]). Feng et al. [40] found that increasing temperature from 20 to 24°C increased growth rate, but only under low light conditions (50 μmol photons m^-2^ s^-1^ versus 400 μmol photons m^-2^ s^-1^). Previous studies using CCMP3266 found growth rates ranging from 0.95 to 1.14 at temperatures between 15°C and 19°C [16, 43, 46]. Langer et al. [16] presented data suggesting a thermal optimum for this strain between 20 and 26°C, though the growth rate was less than half of that found in this study. This higher optimum temperature for growth in temperate phytoplankton has been shown previously, and suggests that these strains may be better able to deal with ocean warming than those found in tropical regions [26, 49].

### Biogeochemical impacts—effect of temperature on CO_2_ dynamics

Temperature could have important biogeochemical implications within surface waters as thermal variations can affect organic and inorganic carbon fixation in coccolithophores [26]. Despite the observed effect of temperature on growth rate in this study, cellular carbon quotas (PIC and POC) were not significantly different across temperature treatments in either strain. Feng *et al*. [40] also found this lack of response in CCMP371 at 20 and 24°C. On the other hand, daily production rates were found to be temperature-dependent though the relationship differed between the two strains. Indeed, daily PIC production in the higher calcifying strain (CCMP3266) showed a positive response to temperature (although this was only detected between 10 and 20°C). In the weaker calcifying strain (CCMP371), daily PIC production appeared to be insensitive to temperature changes between 15 and 20°C despite a large increase in growth rate. This variability in physiological responses to temperature is not unprecedented. For example, Sett et al. [26] found a steady increase in calcification by a Norwegian strain (PLY B92/11) from 15 to 25°C. However, Debodt et al. [50] found PIC production decreased as temperature increased from 13 to 18°C in a strain isolated from the English Channel (RCC1228). Interestingly, Satoh et al. [51] found that calcification increased at colder temperatures (12°C versus 20°C), but only under conditions of phosphate limitation in an *E*. *huxleyi* strain isolated from the Great Barrier Reef of Australia (NIES-837). Daily POC production in this study showed a positive relationship to temperature in both strains with changes likely driven by the large increase in growth rates. This increase in POC production with temperature was similar to that found by Debodt et al. [50] and Sett et al. [26], though they identified a non-linear relationship with temperature, as POC production decreased at 25°C.

Any shift in the relative proportion of inorganic versus organic carbon production has important implications in the ocean’s carbon cycle and possibly in global climate [52]. Specifically, unlike photosynthetic carbon fixation, which consumes CO_2_, the calcification reaction produces CO_2_ by converting bicarbonate ions (HCO_3_^−^) into CaCO_3_. The ratio of PIC:POC (termed the ‘rain ratio’) is important because it controls the direction of CO_2_ fluxes, such that if this ratio is > 1.5, coccolithophores are considered a source of CO_2_ to the environment, and if the ratio is < 1.5, they are considered a sink of CO_2_ [53]. The differences we observe indicate that carbon production during exponential growth by CCMP371 acts as a greater carbon sink than CCMP3266.

### Coccolith production and shedding

The increase in coccosphere size as temperature decreases that was found in both strains in this study is consistent with previous findings from studies using strains isolated from tropical [54], temperate [50], and polar regions [55]. This is also in agreement with findings by Atkinson et al. [56] who show a general inverse relationship between temperature and cell volume in protists, with a 2.5% change per 1°C. In order to test whether changes in cell volume are a result of an increase in the protoplast or an increase in the layers of coccoliths (or both), techniques such as acid-dissolution of coccoliths or focus ion beam sectioning SEM [57] can be used to determine differences between the coccosphere (protoplast surrounded by coccoliths) and the protoplast.

The rate at which coccoliths detach from cells is important for understanding the biogeochemical impact of coccolithophore populations, downward flux of carbon, and aggregation both under bloom and non-bloom conditions. Previous studies have shown coccolith detachment rates to be dependent on growth rate [58–61]. However, these studies were limited to a single strain (88E; strain synonym: CCMP378). Here we provide the first evidence of intraspecific differences in coccolith detachment rates in *E*. *huxleyi*, both in terms of magnitude of detachment as well as temperature-dependent responses. However, caution is necessary to limit interpretation of these data to only comparing differences between strains and temperatures, and not extrapolate to estimate shedding rates in the field. Preservation of samples via fixation and freezing has been shown to reduce cell recovery by 21% compared to live samples in a different strain of *E*. *huxleyi* (RCC174) [62]. This difference is attributed to increased cell lysing, which would overestimate the coccolith:cell ratio. However, since both strains were preserved using identical methods, these results indicate strain-specific differences in either coccolith detachment rates or in cell membrane integrity. As such, these results warrant further investigation into intraspecific differences in coccolith shedding.

The characteristics and quantities of shed coccoliths present in the water column have important implications for carbon fluxes out of the surface ocean. Coccoliths are covered by a layer of acidic polysaccharides, which aid in coccolith formation and attachment to the cell [63–65]. However relatively little is known about the properties of the organic coating and if they may differ across strains. Strains with a ‘less sticky’ organic coating would potentially shed coccoliths more easily than a strain with a ‘stickier’ coating. Coccolithophore-derived calcite has been shown to have a strong effect on formation, size, and sinking velocity of aggregates containing organic matter [66]. In part due to the smaller size and shape of coccoliths, this effect appears to be greater than that of calcite derived from foraminifera, which are less efficient in incorporating organic matter due to their faster sinking rates [25]. Based on the kinetics of particle aggregation, strains with greater rates of coccolith detachment (*i*.*e*., CCMP371) would increase the density of particles in the water compared to lower shedding rates (*i*.*e*., CCMP3266), which could increase the potential formation of aggregates [67].

### Carbonate mineralogy

Across coccolithophores, experimental evidence suggests that the mineralogy of some, but not all, species has likely varied across geological timescales [68]. There have been a number of studies exploring mineralogy in *E*. *huxleyi* using a variety of techniques. Wilbur and Watabe [69] found evidence using X-ray diffraction that *E*. *huxleyi* strain BT-6 (strain synonyms: CCMP373, RCC173) produced calcite with traces of aragonite in nutrient-replete media while strains BT-6 and 92A (strain synonyms: CCMP379, RCC3545) both produced calcite, aragonite, and vaterite in nitrogen-deficient media. However, these results were contradicted by Young et al. [14], who found exclusively calcite while using a similar method. Mann and Sparks [70] identified calcite using electron diffraction and transmission electron microscopy in *E*. *huxleyi* strain 92D (strain synonym: RCC174).

Solid-state nuclear magnetic resonance (NMR) spectroscopy enables the detection and identification of atomic-level ^13^C and ^43^Ca structures and compositions in biogenic carbonates. Specifically, solid-state ^13^C MAS NMR methods have previously been applied to identify and elucidate the local (< 1 nm) structures of carbonate biominerals in a variety of organisms, including coccolithophores, molluscs, and crustaceans [29, 71, 72]. For both types of coccoliths examined here, the single-pulse ^13^C MAS NMR spectra (Fig 6) each exhibit single, intense signals at 168.6 ppm that establish the presence of calcite, which is consistent with X-ray diffraction analyses (S2 Fig) and previous ^13^C NMR results of *E*. *huxleyi* strain CCMP371 [29]. It is noteworthy that the ^13^C MAS spectra contained no other signals in the isotropic ^13^C chemical shift range of 160 to 180 ppm, which is typically associated with ^13^C moieties from other inorganic carbonates [34, 35, 73]. These results establish that calcite predominates in the coccolith biominerals and that there are no other types of inorganic carbonates.

Similarly, the solid-state ^43^Ca MAS NMR spectra (Fig 7) of the coccoliths each exhibit a single narrow signal at 19 ppm that is attributable to calcite, consistent with the ^13^C NMR results above. This is the first time that solid-state ^43^Ca NMR measurements have been conducted on coccolithophore biominerals, especially at the low (0.135%) natural isotopic abundance of ^43^Ca. Other crystalline polymorphs of synthetic calcium carbonate have previously been shown to yield distinct well-resolved ^43^Ca signals at ca. -29 and 10 ppm for aragonite and vaterite, respectively [31, 74], which result from different Ca–O distances and local coordination geometries of ^43^Ca ion sites in the polymorphs [75]. Notably, only the ^43^Ca signal at 19 ppm from calcite is observed in the single-pulse ^43^Ca spectra (Fig 7), even after extensive signal averaging (> 1 x 10^5^ scans), which indicates that there are no detectable quantities of other ^43^Ca moieties. Overall, the preceding ^13^C and ^43^Ca NMR analyses establish that the coccolith biominerals from strains CCMP371 and CCMP3266 examined here are composed principally (if not exclusively) of calcite, with no detectable quantities of other types of inorganic carbonates.

### Ecological implications

A recent emphasis in theoretical community ecology has been placed on the importance of intraspecific variation, specifically on how trait variation may alter interaction strengths, niche breadth, and total population density [76]. Within phytoplankton communities, diversity plays an important role in determining elemental stoichiometry [77], which can fluctuate across space and time. Such variation may have important impacts on nutrient cycling and energy transfer within ecosystems. While there is a rich literature illustrating how environmental parameters (*e*.*g*., light, nutrient availability, temperature) may affect elemental stoichiometry, the magnitude of effect due to intraspecific diversity is less understood (see review [78]).

The ratio of TC:TN reflects the nutritional quality of a cell to grazers, which benefit from selecting prey with higher nitrogen content (and thus lower TC:TN ratio) [79]. Further, strains possessing calcified coccoliths have been shown to reduce grazing rates and grazer growth efficiencies compared to non-calcified strains, potentially due to strain-specific nutrient deficiencies or an alkalization of acidic food vacuoles, which would allow populations of calcified *E*. *huxleyi* to out-grow grazing pressure [80]. In this study, not only did TC:TN differ between strains, but it showed a non-linear relationship with temperature in CCMP3266. These data suggest that CCMP371 would provide a higher-quality food resource to grazers than CCMP3266. The higher levels of calcification found in CCMP3266 could function as an increased grazing deterrent, though this is beyond the limits of this study. Taken as a whole, the magnitude of intraspecific variation found within *E*. *huxleyi* highlights the need to potentially move beyond the traditional species concept to more of a trait-based system for classifying phytoplankton diversity, particularly in light of changing ocean climate. Extrapolating this work to the field would require knowledge of intraspecific diversity in natural populations and their functional responses to their thermal environment.

## Supporting Information

S1 Fig Time series of nutrient concentrations during experimental culturing. Mean (± s.d.) concentrations of nitrate+nitrite (A) and phosphate (B) in each treatment for each strain.
(TIF)

S2 Fig Powder X-ray diffraction patterns of *E*. *huxleyi* strains. Powder X-ray diffraction patterns of *E*. *huxleyi* strains (A) CCMP371 and (B) CCMP3266 acquired at 25°C and using Cu Kα radiation of wavelength 1.54 Å. Each of the patterns exhibit similar intense reflections that are indexable to calcite, as indicated by red markers at the bottom of (B). In addition, several weak reflections indexable to sodium chloride (NaCl) are observed in the pattern in (B), indicated by black markers. Such NaCl species are expected to result from crystallization during freeze-drying of NaCl that is present in the coccolithophore culture medium.
(TIF)

S1 Table Statistical analyses of physiological parameters. Results from three-way ANOVA tests effects of strain (CCMP371 versus CCMP3266), phase (Exponential versus Stationary), and temperature (15°C versus 20°C) on physiological parameters.
(DOCX)

S2 Table Statistical analyses of daily carbon production rates. Results from two-way ANOVA tests of strain (CCMP371 versus CCMP3266) and temperature (15°C versus 20°C) on particulate total, inorganic, and organic carbon during exponential growth.
(DOCX)

S3 Table Statistical analyses of carbon content across 10°C temperature range. Results from two-way ANOVA tests of phase (Exponential versus Stationary) and temperature (10°C versus 15°C versus 20°C) on carbon content in CCMP3266.
(DOCX)
