# Supplementary figures and images for: Intraspecific Differences in Biogeochemical Responses to Thermal Change in the Coccolithophore Emiliania huxleyi
Source: PLoS One. 2016 Sep 1;11(9):e0162313. doi: 10.1371/journal.pone.0162313 (PMC5008731; doi:10.1371/journal.pone.0162313)

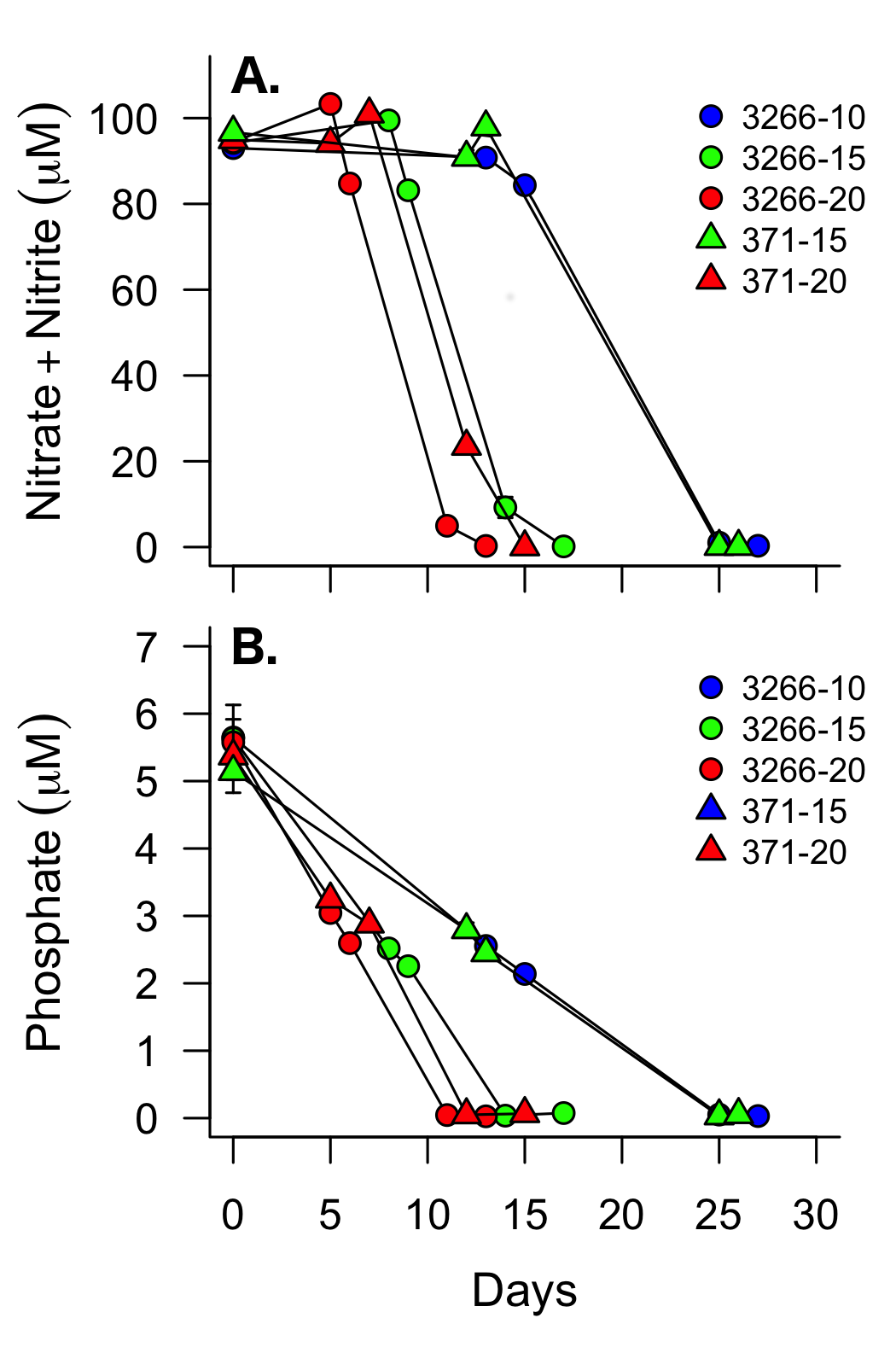

Supplement: S1 Fig — Mean (± s.d.) concentrations of nitrate+nitrite (A) and phosphate (B) in each treatment for each strain. (TIF) [file pone.0162313.s001.tif]

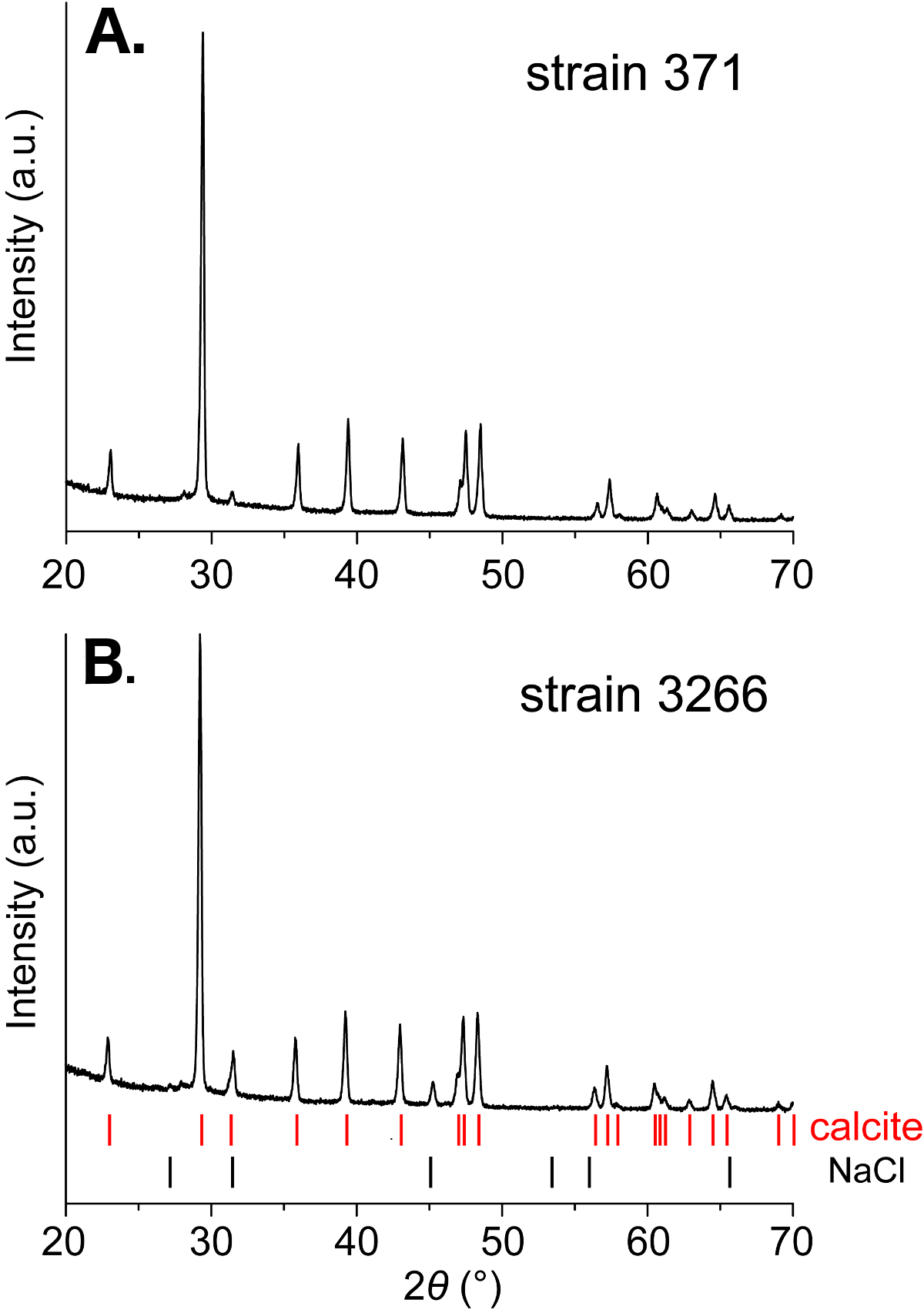

Supplement: S2 Fig — Powder X-ray diffraction patterns of E. huxleyi strains (A) CCMP371 and (B) CCMP3266 acquired at 25°C and using Cu Kα radiation of wavelength 1.54 Å. Each of the patterns exhibit similar intense reflections that are indexable to calcite, as indicated by red markers at the bottom of (B). In addition, several weak reflections indexable to sodium chloride (NaCl) are observed in the pattern in (B), indicated by black markers. Such NaCl species are expected to result from crystallization during freeze-drying of NaCl that is present in the coccolithophore culture medium. (TIF) [file pone.0162313.s002.tif]
