# Supplementary material for: Intraspecific Differences in Biogeochemical Responses to Thermal Change in the Coccolithophore Emiliania huxleyi
Source: PLoS One. 2016 Sep 1;11(9):e0162313. doi: 10.1371/journal.pone.0162313 (PMC5008731; doi:10.1371/journal.pone.0162313)
Supplement: S1 Table — Results from three-way ANOVA tests effects of strain (CCMP371 versus CCMP3266), phase (Exponential versus Stationary), and temperature (15°C versus 20°C) on physiological parameters. (DOCX) [file pone.0162313.s003.docx]

**S1 Table. Statistical analyses of physiological parameters.**

| Variable | Effect | SS | Df | F | *P* |
| --- | --- | --- | --- | --- | --- |
| PIC cell^-1^ | **Strain** | **0.790** | **1** | **5.75** | **0.027** |
|  | Phase | 0.535 | 1 | 3.90 | 0.063 |
|  | Temperature | 0.315 | 1 | 2.30 | 0.146 |
|  | **Strain x Phase** | **1.417** | **1** | **10.31** | **0.005** |
|  | Strain x Temp | 0.014 | 1 | 0.10 | 0.755 |
|  | Phase x Temp | 0.309 | 1 | 2.25 | 0.150 |
|  | S x P x T | 0.009 | 1 | 0.06 | 0.806 |
|  | Residuals | 2.611 | 19 |  |  |
| POC cell^-1^ | **Strain** | **1.003** | **1** | **18.83** | **< 0.001** |
|  | **Phase** | **12.333** | **1** | **231.55** | **< 0.001** |
|  | Temperature | 0.057 | 1 | 1.07 | 0.313 |
|  | **Strain x Phase** | **0.938** | **1** | **17.61** | **< 0.001** |
|  | Strain x Temp | 0.144 | 1 | 2.71 | 0.114 |
|  | Phase x Temp | 0.106 | 1 | 1.98 | 0.173 |
|  | S x P x T | 0.008 | 1 | 0.15 | 0.703 |
|  | Residuals | 1.172 | 19 |  |  |
| TC cell^-1^ | Strain | 0.165 | 1 | 2.76 | 0.113 |
|  | **Phase** | **5.564** | **1** | **93.84** | **< 0.001** |
|  | Temperature | 0.085 | 1 | 1.43 | 0.247 |
|  | Strain x Phase | 0.074 | 1 | 1.25 | 0.278 |
|  | Strain x Temp | 0.056 | 1 | 0.93 | 0.347 |
|  | Phase x Temp | 0.093 | 1 | 1.56 | 0.227 |
|  | S x P x T | 0.015 | 1 | 0.26 | 0.617 |
|  | Residuals | 1.133 | 19 |  |  |
| PIC:POC | **Strain** | **0.556** | **1** | **16.94** | **< 0.001** |
|  | **Phase** | **1.388** | **1** | **42.30** | **< 0.001** |
|  | Temperature | 0.014 | 1 | 0.43 | 0.518 |
|  | **Strain x Phase** | **1.069** | **1** | **32.57** | **< 0.001** |
|  | Strain x Temp | 0.062 | 1 | 1.90 | 0.185 |
|  | Phase x Temp | 0.001 | 1 | 0.04 | 0.845 |
|  | S x P x T | 0.009 | 1 | 0.26 | 0.614 |
|  | Residuals | 0.624 | 19 |  |  |
| TC:TN | **Strain** | **7295.0** | **1** | **31.23** | **< 0.001** |
|  | **Phase** | **2940.3** | **1** | **12.59** | **0.002** |
|  | **Temperature** | **1203.5** | **1** | **5.15** | **0.035** |
|  | **Strain x Phase** | **3154.6** | **1** | **13.50** | **0.002** |
|  | Strain x Temp | 67.6 | 1 | 0.29 | 0.597 |
|  | Phase x Temp | 193.8 | 1 | 0.83 | 0.373 |
|  | S x P x T | 794.4 | 1 | 3.40 | 0.081 |
|  | Residuals | 4438.6 | 19 |  |  |

Results from three-way ANOVA tests of strain (CCMP371 vs. CCMP3266), phase (Exponential vs. Stationary), and temperature (15 ºC vs. 20 ºC) on physiological parameters. PIC, particulate organic carbon; POC, particulate organic carbon; TC, total carbon; TN, total nitrogen. Values in **bold** represent significant effects (*p <* 0.05).
