# Supplementary material for: Intraspecific Differences in Biogeochemical Responses to Thermal Change in the Coccolithophore Emiliania huxleyi
Source: PLoS One. 2016 Sep 1;11(9):e0162313. doi: 10.1371/journal.pone.0162313 (PMC5008731; doi:10.1371/journal.pone.0162313)
Supplement: S2 Table — Results from two-way ANOVA tests of strain (CCMP371 versus CCMP3266) and temperature (15°C versus 20°C) on particulate total, inorganic, and organic carbon during exponential growth. (DOCX) [file pone.0162313.s004.docx]

**S2 Table. Statistical analyses of daily carbon production rates.**

| Variable | Effect | SS | Df | F | *P* |
| --- | --- | --- | --- | --- | --- |
| *P_TC_* | Strain | 13.23 | 1 | 1.03 | 0.340 |
|  | Temperature | 67.22 | 1 | 5.23 | 0.052 |
|  | Strain x Temp | 39.89 | 1 | 3.10 | 0.116 |
|  | Residuals | 102.91 | 8 |  |  |
| *P_PIC_* | **Strain** | **124.70** | **1** | **27.52** | **< 0.001** |
|  | Temperature | 1.36 | 1 | 0.30 | 0.599 |
|  | Strain x Temp | 0.39 | 1 | 0.09 | 0.778 |
|  | Residuals | 36.25 | 8 |  |  |
| *P_POC_* | **Strain** | **56.69** | **1** | **13.17** | **0.007** |
|  | **Temperature** | **49.47** | **1** | **11.49** | **0.009** |
|  | **Strain x Temp** | **48.13** | **1** | **11.18** | **0.010** |
|  | Residuals | 34.43 | 8 |  |  |

Results from two-way ANOVA tests of strain (CCMP371 vs. CCMP3266) and temperature (15 ºC vs. 20 ºC) on daily particulate total, inorganic, and organic carbon production during exponential growth. *P_TC_*, total carbon production; *P_PIC_*, particulate inorganic carbon production; *P_POC_*, particulate organic carbon production. Values in bold represent significant effects (*p <* 0.05).
