# Supplementary material for: Intraspecific Differences in Biogeochemical Responses to Thermal Change in the Coccolithophore Emiliania huxleyi
Source: PLoS One. 2016 Sep 1;11(9):e0162313. doi: 10.1371/journal.pone.0162313 (PMC5008731; doi:10.1371/journal.pone.0162313)
Supplement: S3 Table — Results from two-way ANOVA tests of phase (Exponential versus Stationary) and temperature (10°C versus 15°C versus 20°C) on carbon content in CCMP3266. (DOCX) [file pone.0162313.s005.docx]

**S3 Table. Statistical analyses of carbon content across 10 ºC temperature range.**

| Variable | Effect | SS | Df | F | *P* |
| --- | --- | --- | --- | --- | --- |
| PIC cell^-1^ | **Phase** | **200.67** | **1** | **30.63** | **< 0.001** |
|  | Temperature | 13.44 | 2 | 1.03 | 0.379 |
|  | Phase x Temp | 7.10 | 2 | 0.54 | 0.591 |
|  | Residuals | 117.93 | 18 |  |  |
| POC cell^-1^ | **Phase** | **401.71** | **1** | **76.15** | **< 0.001** |
|  | **Temperature** | **45.97** | **2** | **4.36** | **0.029** |
|  | Phase x Temp | 28.82 | 2 | 2.73 | 0.092 |
|  | Residuals | 94.95 | 18 |  |  |
| TC cell^-1^ | **Phase** | **1170.3** | **1** | **61.31** | **< 0.001** |
|  | Temperature | 108.6 | 2 | 2.85 | 0.084 |
|  | Phase x Temp | 54.9 | 2 | 1.44 | 0.264 |
|  | Residuals | 343.6 | 18 |  |  |
| PIC:POC | **Phase** | **0.171** | **1** | **5.19** | **0.035** |
|  | Temperature | 0.023 | 2 | 0.34 | 0.718 |
|  | Phase x Temp | 0.132 | 2 | 1.99 | 0.165 |
|  | Residuals | 0.594 | 18 |  |  |
| TC:TN | **Phase** | **16.13** | **1** | **5.38** | **0.032** |
|  | Temperature | 20.86 | 2 | 3.48 | 0.053 |
|  | **Phase x Temp** | **25.75** | **2** | **4.29** | **0.030** |
|  | Residuals | 53.95 | 18 |  |  |

Results from two-way ANOVA tests of phase (Exponential vs. Stationary) and temperature (10 ºC vs. 15 ºC vs. 20 ºC) on carbon content in CCMP3266. PIC, particulate organic carbon; POC, particulate organic carbon; TC, total carbon; TN, total nitrogen. Values in bold represent significant effects (*p <* 0.05).
